# In this issue: Volume 115, Issue 11, November 2024

**DOI:** 10.1111/cas.16386

**Published:** 2024-11-03

## Mechanism research of non‐coding RNA in immune checkpoint inhibitors therapy

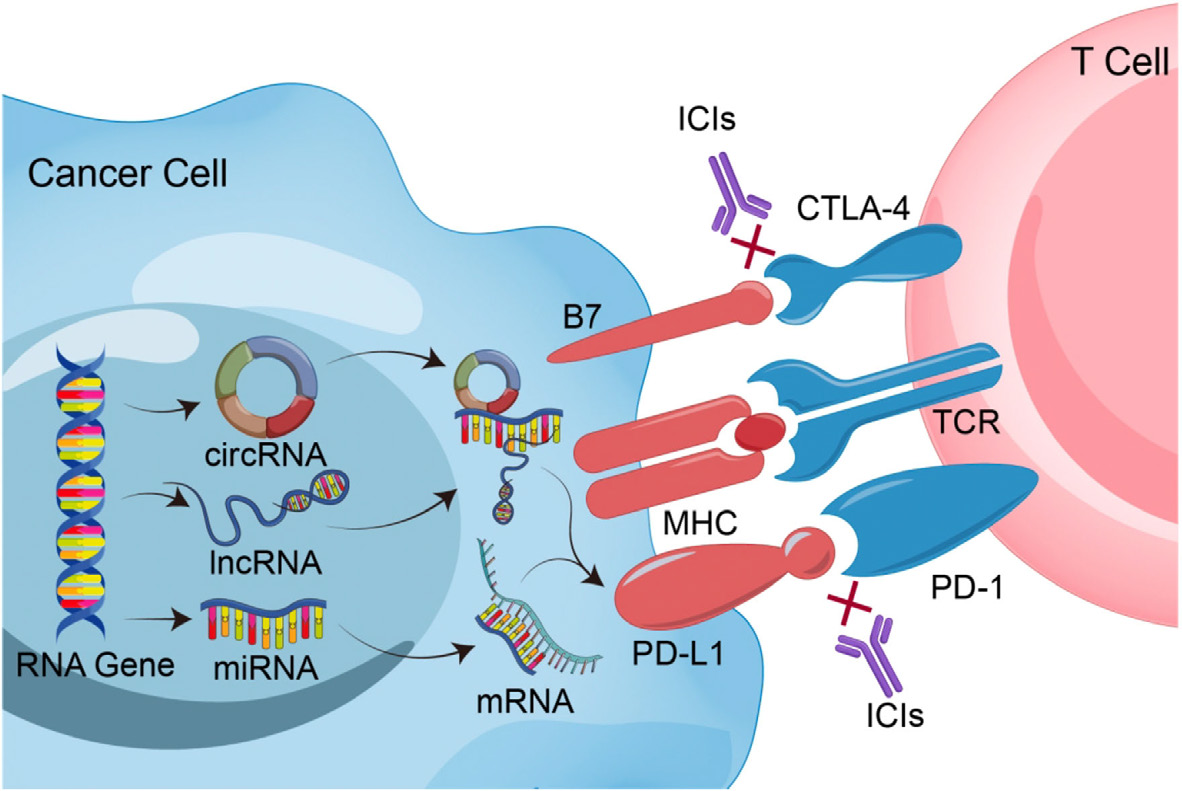

Cells control the amount of proteins that they produce through various post‐transcriptional regulations. One mechanism involves non‐coding RNA molecules (ncRNA), such as micro RNAs (miRNAs), which bind to a target messenger RNA (mRNA) and prevent it from being translated into protein. Cells also produce long non‐coding RNAs (lncRNAs) and circular RNAs (circRNAs) which have other related functions. These molecules are crucial for many of a cell's activities, including signaling to the immune system's T‐cells that it should be left alive.

The hallmark of all cancer cells is their ability to evade normal mechanisms of cell death and multiply uncontrollably. Normally, T‐cells look for specific markers called immune checkpoint proteins to tell apart healthy cells from unhealthy cells. Two important classes of these markers are those that bind to PD‐1 proteins and CTLA‐4 proteins on T‐cells. If these markers are present in enough numbers, T‐cells identify the cell to be healthy and do not kill it.

However, cancer cells produce excessive amounts of immune checkpoint markers to appear healthy and evade the immune system. A recent review by Bian et al. explored how cancer cells regulate these checkpoint markers through ncRNA. Drawing from over 60 studies on ncRNA expression in a wide variety of cancers, the team found four broad mechanisms for immune evasion through checkpoint markers:

- Decreasing the transcription of ncRNAs that normally prevent the production of checkpoint markers.
- Increasing the production of circRNA that blocks miRNA, which would otherwise reduce checkpoint marker levels.
- Increasing the transcription of ncRNA that binds to proteins, which then triggers the production of checkpoint markers.
- Increasing the transcription of lncRNA or circRNA that produce short peptides, which then interfere with immune checkpoint proteins.

In their review, the authors also mention several ncRNAs that could be potential targets for immune checkpoint inhibitory molecules (ICI). ICIs are a type of anti‐cancer drugs that reduce the activity of immune checkpoint markers, usually PD‐1 and CTLA‐4, and allow T‐cells to identify and kill cancer cells. ICIs are becoming popular because they target cancer cells specifically and are much less toxic than chemotherapy or radiotherapy. These findings point toward several new and targeted ICI treatments against a wide variety of cancers, especially those that may not respond to conventional therapies.

https://onlinelibrary.wiley.com/doi/10.1111/cas.16309

## A novel histopathological feature of spatial tumor–stroma distribution predicts lung squamous cell carcinoma prognosis

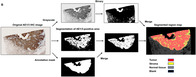

Squamous cell carcinoma (SCC) is a major type of lung cancer, making up 15%–20% of cases. Despite treatment advances, SCC prognosis after surgery remains poor, with recurrence rates between 25% and 40%. A key challenge in SCC is the lack of reliable pathological markers to predict tumor behavior and patient outcomes. Unlike adenocarcinoma, which has established prognostic markers, SCC has limited options. While some features, like tumor budding, have been suggested as predictors of poor outcomes, none are widely accepted.

In this study, Taki et al. explored a new method to analyze lung cancer tissues, focusing on the spatial arrangement of cancer cells and the supportive tissue surrounding them, known as stroma. They examined tissue samples from 132 patients with SCC, using a machine learning approach called Simple Linear Iterative Clustering (SLIC). Their research introduced the Spatial Tumor‐Stroma Distribution Index (STSDI), which incorporates a spatial form of Shannon's entropy and Euclidean distance to assess how cancer cells and stroma are organized. The study found that lower STSDI values were associated with worse patient outcomes, including higher rates of recurrence and lower survival rates, highlighting the importance of spatial distribution in understanding lung cancer behavior.

The findings revealed that compared to patients with high STSDI, patients with low STSDI had significantly lower 5‐year recurrence‐free survival (49.5% vs. 76.2%) and 5‐year disease‐specific survival (53.6% vs. 81.5%). Additionally, low STSDI was linked to more aggressive tumor growth patterns. The research demonstrated that traditional measures, like tumor‐stroma ratio, were less effective in predicting outcomes than the STSDI, which accounts for spatial distribution.

Overall, this innovative approach offers pathologists a valuable tool for assessing lung cancer tissues, helping to predict tumor behavior and guide treatment decisions for patients with SCC. The study emphasizes the need to consider not just the quantity of cancer cells and stroma, but also their spatial relationships, providing a new perspective in cancer prognosis and management.

https://onlinelibrary.wiley.com/doi/10.1111/cas.16244

## Improved platelet separation performance from whole blood using an acoustic fluidics system

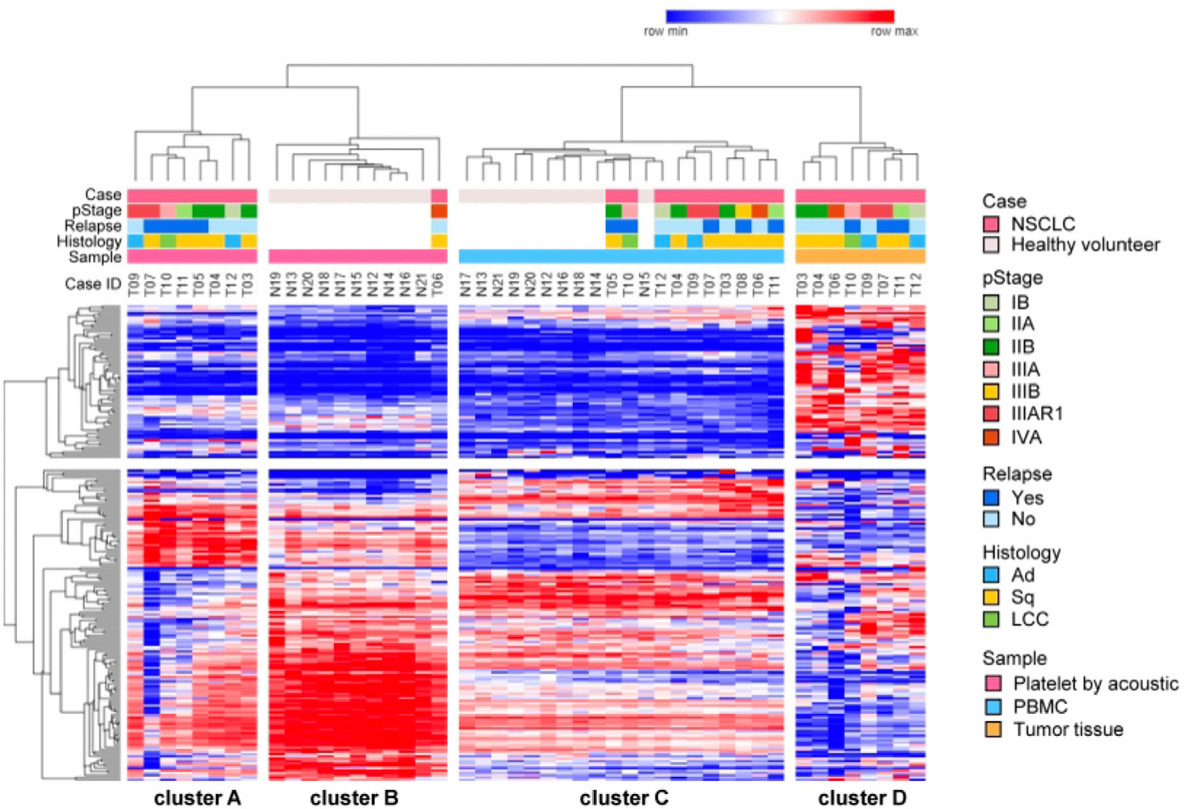

Platelets are best known for their functions in blood clotting, but they can also act as key indicators of cancer. Timely detection of cancer and knowing its exact type are crucial for effective cancer treatment. In non‐small cell lung cancer (NSCLC), the changes in platelets' genetic make‐up could indicate the presence of cancer.

To study the differences in the expression levels of genes in platelets, they need to be separated from the red and white blood cells. For many years, people have been using centrifugation‐based methods where blood or plasma is spun at a certain speed to separate platelets based on the density of the particles. These methods are labor‐intensive.

In a newly published study, Sakai et al. propose an acoustic method wherein ultrasound is used for platelet separation. In this method ultrasound is used to generate waves inside a tiny channel. The waves affect the movement of blood cells in the channel, leading to their separation based on density. Platelets are the lightest of all blood cells and can be separated in this manner.

The researchers conducted a study to test their hypothesis using blood samples from 10 healthy individuals and 10 individuals with NSCLC. They found that platelet separation by the acoustic or ultrasound method yielded greater numbers. To study gene expression, RNA needs to be isolated from cells. Analysis showed that the platelets obtained from the ultrasound method provided superior quality RNA. The researchers also studied gene expression patterns and deduced that platelets from patients with NSCLC had profoundly different gene expression patterns compared to platelets from healthy individuals.

The acoustic method for platelet separation can therefore be developed into an automated alternative to traditional labor‐intensive methods. As the findings suggest, the gene expression profile of the platelets isolated in this manner can indeed be used to detect cancer and understand its mechanism, highlighting further advantages of this advanced technique.

https://onlinelibrary.wiley.com/doi/10.1111/cas.16337

